# Wearable Bioimpedance-Based Deep Learning Techniques for Live Fish Health Assessment under Waterless and Low-Temperature Conditions

**DOI:** 10.3390/s23198210

**Published:** 2023-09-30

**Authors:** Yongjun Zhang, Longxi Chen, Huanhuan Feng, Xinqing Xiao, Marina A. Nikitina, Xiaoshuan Zhang

**Affiliations:** 1 School of Information Engineering, Shandong Youth University of Political Science, Jinan 250103, China; 190057@sdyu.edu.cn (Y.Z.);; 2 Smart Healthcare Big Data Engineering and Ubiquitous Computing Characteristic Laboratory, Universities of Shandong, Jinan 250103, China; 3 New Technology Research and Development Center of Intelligent Information Controlling, Universities of Shandong, Jinan 250103, China; 4 College of Engineering, Beijing Laboratory of Food Quality and Safety, China Agricultural University, Beijing 100107, China; 5 V.M. Gorbatov Federal Research Center for Foods Systems of RAS, 109316 Moscow, Russia

**Keywords:** live fish health monitoring, waterless and low-temperature conditions, deep learning, wearable bioimpedance monitoring, stress evaluation

## Abstract

(1) Background: At present, physiological stress detection technology is a critical means for precisely evaluating the comprehensive health status of live fish. However, the commonly used biochemical tests are invasive and time-consuming and cannot simultaneously monitor and dynamically evaluate multiple stress levels in fish and accurately classify their health levels. The purpose of this study is to deploy wearable bioelectrical impedance analysis (WBIA) sensors on fish skin to construct a deep learning-based stress dynamic evaluation model for precisely estimating their accurate health status. (2) Methods: The correlation of fish (turbot) muscle nutrients and their stress indicators are calculated using grey relation analysis (GRA) for allocating the weight of the stress factors. Next, WBIA features are sieved using the maximum information coefficient (MIC) in stress trend evaluation modeling, which is closely related to the key stress factors. Afterward, a convolutional neural network (CNN) is utilized to obtain the features of the WBIA signals. Then, the long short-term memory (LSTM) method learns the stress trends with residual rectification using bidirectional gated recurrent units (BiGRUs). Furthermore, the Z-shaped fuzzy function can accurately classify the fish health status by the total evaluated stress values. (3) Results: The proposed CNN-LSTM-BiGRU-based stress evaluation model shows superior accuracy compared to the other machine learning models (CNN-LSTM, CNN-GRU, LSTM, GRU, SVR, and BP) based on the MAPE, MAE, and RMSE. Moreover, the fish health classification under waterless and low-temperature conditions is thoroughly verified. High accuracy is proven by the classification validation criterion (accuracy, F1 score, precision, and recall). (4) Conclusions: the proposed health evaluation technology can precisely monitor and track the health status of live fish and provides an effective technical reference for the field of live fish vital sign detection.

## 1. Introduction

The monitoring and evaluation of live fish stress levels is generally considered an effective approach to acquiring their health status in the aquatic food industry [1]. When fish are subjected to extreme environmental stress, such as hypoxia, low temperatures, and water scarcity, it may arouse a decrease in their metabolic rate and varying degrees of stress response to maintain homeostasis in their bodies [2,3]. Compared to normal conditions, fish undergo significant changes in their physiological and biochemical processes, such as hormone secretion and material energy metabolism during stress reactions, which can directly affect fish health and have adverse effects on their muscle qualities. Furthermore, the physiological variations of live fish experience nonlinear and dynamic life decline with various stress interferences, exhibiting multi-scale stress changes in individuals [4,5].

At present, a series of traditional analytical techniques have been employed to obtain accurate stress level evaluations in live fish quality [6,7]. Some research shows that the serum cortisol, lactate, and blood glucose are the most important factors of fish stress indicators, and their contents significantly increase under low temperatures and anhydrous conditions [8]. Currently, the commonly adopted approaches to fish stress testing mainly focus on blood biochemical tests; however, accurate testing results come at the cost of invasive testing, which may generate indefinite results owing to the changes in the original inspection conditions. The current fish stress detection methods and their pros and cons are categorically presented in Table 1.

Based on the above analysis, it is urgent to design a wearable and non-invasive measuring and modeling method for evaluating original fish stress variations and acquiring their declining health patterns, which will enable the precise observation of fish health without reducing their vitality or survival quality. In recent studies, some researchers have focused on WBIA-related detection for aquatic food quality or biological health levels. Fan et al. [18] proposed a prediction method for the non-destructive freshness measurement of rainbow trout during ice storage using impedance technology. Their findings exhibited high correlations with the key quality indexes, such as hardness, K-value, and rigor mortis. Other scholars have reported that bioimpedance signals effectively reflect the ATP-related compound variations in fish and conveniently assess their freshness [19]. Classifying fish freshness using the peaks in the electrochemical impedance spectroscopy morphological characteristic curves has made great improvements to the classification accuracy [20]. Curtis et al. [21] experimentally verified the effects of five parameters related to fish handling on a momentary body condition index (phase angle) measured using a suitable BIA-based approach for quickly detecting an individual’s conditions. As mentioned above, numerous studies have shown that the health status of live fish can be accurately measured by the levels of blood stress and antioxidant substances [22]. Some scholars have also utilized electrochemical impedance spectroscopy (EIS) and quartz crystal microbalance (QCM) for the detection of three fish hormones, cortisol, insulin-like growth factor 1 (IGF-1), and vitellogenin [23]. They discussed prospective applications to understand fish physiology from the aspect of hormone measurements. Moreover, BIA is a common tool in human health and physiology assessment that has recently been adaptively applied to fish and wildlife. It can provide accurate estimates of body composition in fish, such as water, protein, fat, and percent dry mass [24,25,26].

Except for the above BIA-based fish health measurement, the image-processing application of camera images, microscopic images, spectral images, ultrasound images, and fluorescence images has provided non-destructive, automatic, rapid, and real-time approaches to fish health status evaluation [27]. For in vivo glucose monitoring in animal studies, millimeter-wave sensing technology is utilized in the 58–62 GHz frequency range for stress variations in pigs [28]. Microwave resonators can also potentially be used for an individual’s blood glucose level. This is reflected in the correlation coefficients between the glycemia and the consistent measuring magnitude for continuous stress analysis [29]. Additionally, some scholars have developed a variety of biosensors that can be used to measure the target factors of fish health by combining bio-catalysis technology with electronic technology so that the indicators of fish health can be measured quickly and easily [30]. The non-invasive detection of glucose content was also proposed using a density-transformed microstrip patch antenna [31]. In addition, a wireless biochemical sensing system for the continuous monitoring of glucose in flounder has been developed. When the sensor is placed into the sclera, changes in the glucose concentration can be monitored in real time [32]. Some scholars also used infrared reflection technology to automatically track fish to reduce human interference and improve the accuracy of fish behavior detection [33].

Nevertheless, few current studies are focused on the non-destructive detection of multiple factors of stress for live fish quality classification. The research gaps are that critical physiological stress indicators cannot be simultaneously measured by one probe unit, such as an exclusive biosensor, image-grabbing device, or electromagnetic spectrometer, which makes it difficult to simultaneously acquire multiple stress factors in fish and comprehensively determine their health classification. Therefore, the significance of our study is that we applied WBIA-based key stress detection with a multifrequency combination for fish vitality evaluation under adverse conditions. Furthermore, the multiple stress measurements also pave the way for optimizing live fish quality control using deep learning estimation modeling for precise stress acquisitions and reasonable stress-based health classifications.

In this study, we first tested and calculated the relationship between fish muscle nutrients and key stress factors to set the different weights of the key stresses for the final fish health classifications. Then, WBIA signals on the fish skin were filtered and selected, which is more related to the stress trends. Afterward, the deep learning-based stress factors evaluation approaches were established for estimating precise stress variations. Ultimately, we conducted fuzzy membership function-based health classification by weighted and normalized stresses to improve the accuracy of fish health status predictions.

This paper is organized as follows. Section 2 summarizes the related concepts and theories of wearable WBIA-based stress estimation and health classification approaches. Next, the experimental scheme is presented, and the WBIA-based stress monitoring system is explained. Then, the fish health assessment approach is described using the obtained dynamic stress variations and stress-based fuzzy health classification. Section 3 and Section 4 verify and discuss the experimental results to obtain a final integrated conclusion.

## 2. Materials and Methods

### 2.1. Experimental Scheme for Health Evaluation

#### 2.1.1. Experimental Equipment

The test equipment is listed as follows: HBS-1096 enzyme marker, Nanjing DeTie Biotechnology Co., Ltd., Nanjing, China; FYL-7S-431L Refrigerator (0–20 °C), Beijing FuYi Electrical Appliance Co., Ltd., Beijing, China; LP-31 aquarium three-in-one gas exposure machine, Shenzhen Xingrisheng Industrial Co., Ltd., Shenzhen, China; 5810R high-speed freezing centrifuge, Shanghai Aice Electronic Technology Co., Ltd., Shanghai, China; CK-2 Fish Tank Refrigerator, Guangzhou Chengke Electronic Technology Co., Ltd., Guangzhou, China; ELISA detection kit (fish blood indexes: glucose, cortisol; fish muscle indexes: lactate, glycogen), Shanghai Coibo Biotechnology Co., Ltd., Shanghai, China; ultraviolet-visible spectrophotometer (VIS-721N), Shanghai YiDian Scientific Instrument Co., Ltd., Shanghai, China; LC-8000 ultra high-performance liquid chromatography instrument, Beijing JITIAN Instrument Co., Ltd., Beijing, China.

#### 2.1.2. Experimental Scheme

**Figure 1:**
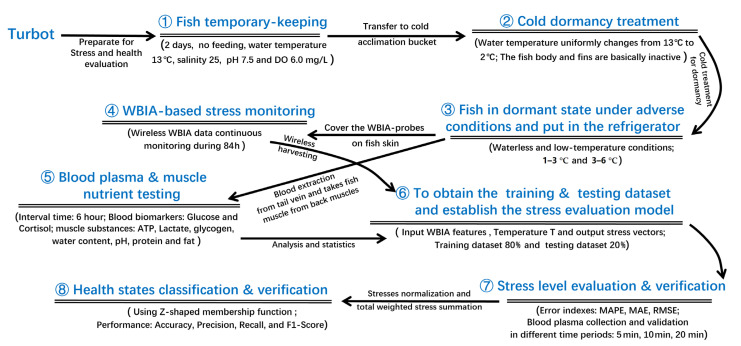
The experimental scheme for live fish health assessment under waterless and low-temperature conditions.

The experimental scheme for live turbot health monitoring, evaluation, and verification is illustrated in Figure 1.

In this study, we generally defined the fish size features (weight, length, and width) into three categories: small size (weight: 957.1 g ± 12 g; length: 34.4 cm ± 3 cm; width: 25.4 cm ±2.5 cm); medium size (weight: 977.4 g ± 10 g; length: 37.5 cm ± 3 cm; width: 27.9 cm ± 2.5 cm); large size (weight: 987.4 g ± 10 g; length: 39.5 cm ± 3 cm; width: 29.2 cm ± 3 cm). The experimental subjects were medium-sized turbot temporarily bred in a prepared seawater tank for 2 days without feeding. The water was kept at an average water temperature of 13 °C, salinity of 25%, pH of 7.5, and an average dissolved oxygen (DO) content of 6.0 mg/L. Next, the fish were transferred to a cold taming bucket for cold dormancy treatment by gradually reducing the water temperature from 13 °C to 2 °C at a rate of 2 °C/h. After that, the dormant turbot were gently collected and put on a wet sponge in a plastic bag. One piece was put in each plastic bag, and the air was emptied. Then, the bags were filled with pure oxygen, tightened, and stacked in the refrigerator at 1–3 °C and 3–6 °C for the waterless and low-temperature physiological stress monitoring and verification tests.

At first, we determined the near-death time point to clearly identify the health decline process. At this stage, 25 fish were prepared to accomplish this verification. We observed the subjects every hour (recording the respiratory rate (times/min), gill flapping amplitude, body surface color, converged fin angle, behavior changes, and duration (hours)) using video recognition technology. When the survival rate was less than 60% in these adverse conditions, we ended the live status observation to record the near-death time point by judging the gills that remained motionless and unresponsive to stimulation, as a key reference indicator to measure the fish health levels.

Meanwhile, WBIA-based stress monitoring and verification was carried out as follows: Firstly, 35 turbot were temporarily bred in a prepared seawater tank for 2 days without feeding. Five fish were placed in each layer, and the sides of their bodies were covered with wireless WBIA electrodes, with a total of eight layers, for anhydrous low-temperature vitality monitoring. At 0, 12, 18, 24, 48, 60, 72, and 84 h, five live fish samples were taken for subsequent testing and analysis. At the checking time points, we used a 2 mL disposable syringe to draw blood from the tail vein. Whole blood without anticoagulant was transferred to a 2.0 mL centrifuge tube and placed in a refrigerator at 4 °C for 2 h. Then, the plasma was prepared for centrifuge treatment, which was set at 1200× *g* (centrifugal force) for 10 min. Finally, the serum was collected in a 1.5 mL centrifuge tube and stored at −80 °C until analysis. Afterward, we slaughtered the turbot and inserted a pH probe to measure the muscle pH at the above assigned time points. The back muscles of the turbot (5 in each group) were sampled and placed on ice, quickly removed, and stored in a refrigerator at −20 °C for testing the other muscle indicators. Finally, the muscle ATP-related compounds were analyzed using high-performance liquid chromatography. Lactate and glycogen kits (CB10190-Fi, Shanghai Coibo Biotechnology Co., Ltd., Shanghai, China) were applied to determine the lactic acid and glycogen in the muscles of the turbot, and we used an ultraviolet-visible spectrophotometer (VIS-721N) to obtain the readings.

### 2.2. Health Monitoring System

#### 2.2.1. Wearable Bioelectrical Bioimpedance Analysis (WBIA)

Regarding dynamic stress factor detection, the WBIA module was deployed, which uses multiple probes covering the fish skin to continuously measure the fish stress levels and evaluates their degree by analyzing the measured fish skin bioimpedance signals [34]. During the measurement, the WBIA sensing module returns the real value R and imaginary value I of the impedance Z to be measured at each sweep frequency point, and its impedance amplitude M and phase θ are calculated by Equations (1) and (2).

(1) $$M=\sqrt{R^{2}+I^{2}}$$

(2) $$\theta =arctan\left(I/R\right)$$

After calculating the amplitude M of measuring the bioimpedance and obtaining the gain factor K using the known resistance (such as 1 kΩ, 2 kΩ, etc.), the impedance value Z is computed by Equation (3):

(3) $$Z=\frac{1}{M\times K}$$

In Section 2.3.3, we take the impedance value Z and its phase θ as the input features for obtaining the predicted stress status. 

#### 2.2.2. Data Preprocess

In this study, the Romanovsky criterion was utilized to remove large errors to improve the accuracy of the WBIA data acquisitions [35]. This criterion can detect whether the raw series contains a gross error and remove suspicious measurement values, which satisfies Equation (4).

(4) $$\left\{\begin{array}{l} \left|x_{i}-x_{p}\right|>K\sigma \\ \sigma =\sqrt{\frac{\sum_{i=1,i\neq j}^{n}\left(x_{i}-x_{p}\right)}{n-2},}x_{p}=\frac{1}{n-1}\sum_{i=1,i\neq j}^{n}x_{i} \end{array}\right.$$

where the criterion distinguishes gross errors based on the actual error distribution range of the t-distribution. Set a set of monitoring data $\left\{x_{1},x_{2},\ldots ,x_{i},\ldots x_{n}\right\}$ and assume the measurement value $x_{i}$ are suspicious data. If suspicious data are removed, $x_{i}$ needs to meet $\left|x_{i}-x_{p}\right|>K\sigma$, where $x_{p}$ is the average value of the remaining data after removing the data; Kσ is the critical value; K is the t-distribution test coefficient, and σ is the experimental standard deviation of the remaining measurement data.

Savitzky–Golay smoothing is applied to a data series to smooth it and improve the accuracy of the data without changing the signal trend or width [36]. This tool is illustrated in Equation (5) and implemented through the process of convolution, which involves fitting a continuous subset of adjacent data points with a low-order polynomial using the linear least squares method.

(5) $$x_{k,\;\mathrm{smooth}\;}=\bar{x_{k}}=\frac{1}{H}\sum_{i=-w}^{+w}x_{k+i}h_{i}$$

where $h_{i}/H$ is the smoothing coefficient obtained by fitting a polynomial using the least squares method. w is the width of the windows.

#### 2.2.3. Stress Weight Allocation and WBIA Feature Selection

Generally, the nutrients in fish muscle gradually decrease, which may be caused by a decline in the health condition of the fish [37]. Among the nutrients, fat and crude protein are important energy sources for fish, and their consumption process may accelerate in stressful conditions. Muscle glycogen reserves are also mobilized to provide energy. In addition, lactate accumulation in the muscle will lead to a decrease in the muscle pH and reduce the meat quality. ATP also provides the energy necessary to maintain fish’s normal life activities. Furthermore, studying the hormone levels of stressed fish is crucial for evaluating their health status [38]. Changes in the external environment will activate the hypothalamic–pituitary renal tissue system of fish, leading to the production of a large number of hormones to maintain their physiological balance and achieve longer survival. In this work, the values of the stress factors at different time points are denoted as the following matrix in Equation (6):

(6) $$S_{n\times t}=\left[\begin{array}{l} s_{11},s_{12},\cdots ,s_{1t}\\ s_{21},s_{22},\cdots ,s_{2t}\\ \cdots \cdots \cdots \cdots \cdots \cdots \\ s_{n1},s_{12},\cdots ,s_{nt} \end{array}\right]$$

where $S_{n\times t}$ is the fish stress discrete detection at time t, and n is the number of stress factors.

Grey relationship analysis (GRA) is a multi-factor statistical analysis method [39]. In terms of these tools, we determined which stress indicators were more related to fish edible quality, and which factors were relatively weak. GRA is formally expressed as Equation (7).

(7) $$\zeta_{i}(k)=\frac{min_{i}min_{k}\left|x_{0}(k)-x_{i}(k)\right|+\rho \cdot max_{i}max_{k}\left|x_{0}(k)-x_{i}(k)\right|}{\left|x_{0}(k)-x_{i}(k)\right|+\rho \cdot max_{i}max_{k}\left|x_{0}(k)-x_{i}(k)\right|}$$

where the comparison series is $x_{i}$. The reference series is $x_{0}$. ρ is the resolution coefficient, which is in the range of (0, 1). The smaller the value of ρ, the greater the resolution. $\zeta_{i}$ represents the correlation degree between the data of the *i*-th indicator and the evaluation result. Thus, the grey correlation weight of the *i*-th indicator is calculated using the expression $w_{i}=\zeta_{i}/\sum_{i=1}^{n}\zeta_{i}$. The weight of certain stress factors is expressed as follows: $W=\left[w_{1},w_{2},\cdots ,w_{n}\right]$.

To better analyze the WBIA features and the stress variations, the maximum information coefficient (MIC) was selected, as it can more sensitively determine their depth correlation compared to the Pearson, Spearman, and other linear correlation analysis methods due to its universality and robustness [40]. Its value range is between 0 and 1, and higher values indicate a stronger correlation. The whole process of MIC calculation is divided into three steps. (1) Given *i* and *j*, the scattergram composed of *X*, and *Y* is gridded into *i* columns and *j* rows, and the maximum mutual information value is calculated; (2) the MIC value is normalized; (3) the MIC value is concisely expressed as Equation (8).

(8) $$MIC\left[x;y\right]=\underset{\left|X\right|\left|Y\right|<B}{max}\frac{I\left[X;Y\right]}{log_{2}\left(min\left(\left|X\right|,\left|Y\right|\right)\right)}$$

In the above equation, I is mutual information. There are a number of partitioned grids in the x, y directions, essentially the grid distribution, and *B* is the variable. Generally, the size setting of *B* is about 0.6 times the data volume. In this work, we used this correlation analysis tool to obtain highly related WBIA features as the input parameters for the next more precise deep learning-based stress level estimation. 

#### 2.2.4. WBIA-Based Health Monitoring System

**Figure 2:**
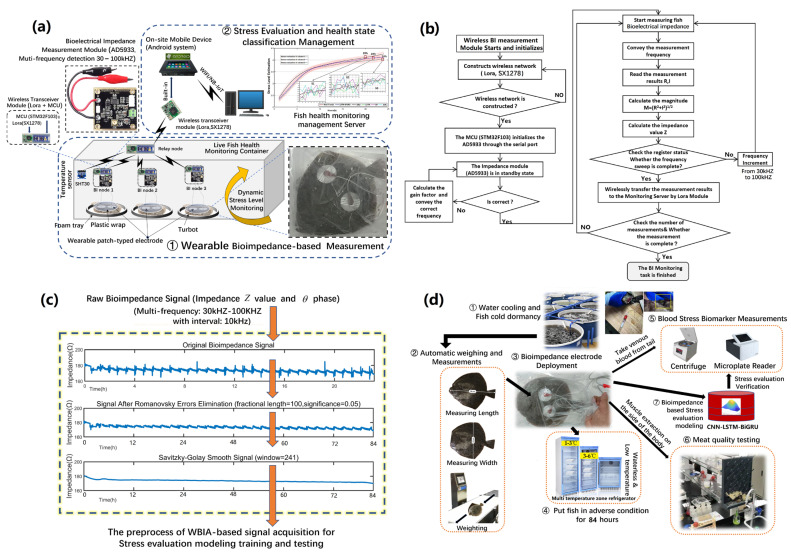
Semantic description of wireless WBIA-based stress monitoring and evaluation process: (**a**) WBIA system architecture for live fish stress detection and health status classification; (**b**) working procedure of WBIA signal monitoring and wireless harvesting; (**c**) preprocessing of the WBIA signal acquisition; (**d**) fish stress biomarker acquisitions, testing, its weight allocation and evaluation model verification in waterless and low-temperature conditions.

In this work, a health monitoring system was designed for the continuous stress monitoring of live fish health conditions. As shown in Figure 2a, the fish stresses were evaluated by a wireless WBIA system.

Firstly, the WBIA module (AD9533, Analog Devices, Inc., Wilmington, MA, USA) sampled the WBIA signals (bioimpedance and phase) under different frequencies from 30 kHZ to 100 kHZ and combined the temperature (SHT31, Sensirion, Stäfa, Switzerland) data series with packaging in an STM32F103 micro-controller (STMicroelectronics, Geneva, Switzerland). Then, the data were harvested by Lora (Semtech, Camarillo, CA, USA) in live fish monitoring containers. Afterward, these data were transmitted to an on-site mobile device for data temporary storage and preprocessing. Finally, the grouped data in the previous nodes were collected and merged in the stress evaluation modeling server for the fish health comprehensive evaluation and classification. In Figure 2b, the working procedure of multi-frequency WBIA signal harvesting is illustrated in a flow chart as a guide for the monitoring procedure. As shown in Figure 2c, the raw WBIA signals were preliminarily processed by the Romanovsky criterion to remove the large error deviations. Next, the Savitzky–Golay filter further smoothed the signals and constructed the training data set for the stress evaluation modeling. As shown in Figure 2d, fish stress biomarkers were deliberately acquired, tested, weight allocated, and verified for health status classification modeling.

### 2.3. Health Level Assessment Modeling

#### 2.3.1. Health Level Calculations

In this test scenario, the live fish health levels were roughly divided into five levels: strong live level (SLL), medium live level (MLL), basic live level (BLL), weak live level (WLL), and death status (DS). The turbot were in cold dormant conditions with the initial filled oxygen level (95%) and a time duration of about T hours in waterless and low-temperature conditions (1–3 °C and 3–6 °C). The initial low-temperature dormancy and near-death time points are important reference indicators for monitoring the health of live fish under adverse conditions. Thus, the key behavior indexes of the respiratory rate (times/min), gill flapping amplitude, body surface color, converged fin angle, survival rate, and behavior changes were the evaluation basis for the health-level classifications. Therefore, the health levels were measured by the degree of fish nutrient decline from the strong live level to the death status, divided according to the above-mentioned key behavioral indexes (in Section 3.4) and expressed by Equation (9).

(9) $$\left\{\begin{array}{c} HS=\underset{\left[T_{low},T_{high}\right]}{dist}\left(TNu_{0},TNu_{j}\right)\\ TNu_{0}=\frac{1}{H}\sum_{i=1}^{H}\left\{norm\left[Nu_{i}\left(t_{0}\right)\right]\right\}\\ TNu_{j}=\frac{1}{H}\sum_{i=1}^{H}\left\{norm\left[Nu_{i}\left(t_{j}\right)\right]\right\} \end{array}\right.$$

where $\left[T_{low},T_{high}\right]$ is the temperature zone. H is the number of nutrients. $TNu_{0}$ ($TNu_{0}$ = 1) is the initial normalized value of all nutrients. $TNu_{j}$ (0 ≤$\;TNu_{j}$ < 1) is the normalized value of all nutrients at time j(j≤T). HS is the health score that is calculated by the Euclidean distance between $TNu_{0}$ and $TNu_{j}$ (HS is in [0, 1]). 

For comprehensive health level identification, the total stress value is a biomarker-based judgment-dependent indicator. Its value is normalized to a range of 0 to 1 by summing each weighted and normalized stress value. For a certain fish size, the total fish stress value $S_{total}^{t}$ is calculated as shown in Equation (10).

(10) $$S_{total}^{t}=\sum_{i=1}^{n}\left\{W_{i}\times norm\left(S_{i}\left(t\right)\right)\right\}$$

where $norm\left(S_{i}\left(t\right)\right)$ is the normalization of a certain stress factor $S_{i}$ at time t. n is the number of stress factors. W is the stress factor weight deduced by the GRA. 

In experiments, the nutrient decline trends and stress factor variations are presented by an inversely proportional relationship, which is generally described as Z-shaped functions by setting the different adjustment coefficients [41]. The health quality level classification model is expressed as a fuzzy mapping calculation using the Z-shaped membership function (ZMF), which is given by the following Equation (11):

(11) $$HQ\left(S_{total}^{t},W,a,b\right)=\left\{\begin{array}{ll} 1, & S_{total}^{t}\leq a\\ 1-2{\left(\frac{S_{total}^{t}-a}{b-a}\right)}^{2}, & a\leq S_{total}^{t}\leq \frac{a+b}{2}\\ 2{\left(\frac{S_{total}^{t}-b}{b-a}\right)}^{2}, & \frac{a+b}{2}\leq S_{total}^{t}\leq b\\ 0 & S_{total}^{t}\geq b \end{array}\right.$$

where $HQ\left(S_{total}^{t},W,a,b\right)$ is the health quality mapping function. The result of this fuzzy function is [0, 1]. a and b are the adjustment coefficients that will make the ZMF fit well with the health score and the corresponding total normalized stress values. For well-fitting health scores and stress values, the two ZMF curves were used to enclose the detection points from alive to near death. Therefore, the ZMF-based health mapping curve was calculated using the mean value of the two enclosing curves’ parameters as its adjustment coefficients. In addition, the HQ calculations are more specifically discussed and instantiated in Section 3.4.

#### 2.3.2. Deep Learning Models

In this section, deep learning-based stress estimation methods are introduced and discussed for obtaining stable and precise stress variations. Previous studies have shown that one-dimensional CNN has strong feature extraction capabilities in temporal data processing [42]. Depending on the monitored historical changes of WBIA signals, gated recurrent units (GRUs) allow each recurrent unit to be adaptively captured independently at different time scales to better evaluate fish health status [43]. GRUs also combine the input gate and forgetting gate of long short-term memory (LSTM) into an update gate, and change the output gate into a reset gate, which makes it have a simpler structure. Thus, it is easier to train and update its hidden status with less computation [44]. However, unidirectional LSTM and GRU neural networks have problems, such as the insufficient utilization of data information. Considering the above issues, the BiGRU model was reasonably introduced in this work to capture the two-way information flow of the time series-based stress features and more accurately learn the dynamic trends of the stresses. The BiGRU model consists of four parts, an input layer, forward propagation layer, backward propagation layer, and output layer, which are divided into two processes: forward propagation and backward propagation. It trains each time series through two GRUs, forward and backward. The calculation of the hidden status at time *t* is shown in Equation (12).

(12) $$h_{t}=\left\{\begin{array}{c} \vec{h_{t}}=f\left(\vec{x_{t}^{\left(u\right)}},\vec{h_{t-1}};{\vec{\Theta }}_{\mathrm{BiGRU}}\right)=\left\{\begin{array}{l} \vec{z_{t}}=\sigma \left({\vec{W}}_{xz}\vec{x_{t}^{u}}+{\vec{W}}_{hz}\vec{h_{t-1}}+\vec{b_{z}}\right),\vec{r_{t}}=\sigma \left({\vec{W}}_{xr}\vec{x_{t}^{u}}+{\vec{W}}_{hr}\vec{h_{t-1}}+\vec{b_{r}}\right)\\ \tilde{h_{t}}=tanh\left({\vec{W}}_{xh}\vec{x_{t}^{u}}\right)+\vec{U}\left(\vec{r_{t}}⊙\vec{h_{t-1}}\right),\vec{h_{t}}=\left(1-\vec{z_{t}}\right)⊙\vec{h_{t-1}}+\vec{z_{t}}⊙\tilde{h_{t}} \end{array}\right.,forward\\ \overset{\leftarrow }{h_{t}}=f\left(\overset{\leftarrow }{x_{t}^{\left(u\right)}},\overset{\leftarrow }{h_{t-1}};{\overset{\leftarrow }{\Theta }}_{\mathrm{BiGRU}}\right)=\left\{\begin{array}{l} \overset{\leftarrow }{z_{t}}=\sigma \left({\overset{\leftarrow }{W}}_{xz}\overset{\leftarrow }{x_{t}^{u}}+{\overset{\leftarrow }{W}}_{hz}\overset{\leftarrow }{h_{t-1}}+\overset{\leftarrow }{b_{z}}\right),\overset{\leftarrow }{r_{t}}=\sigma \left({\overset{\leftarrow }{W}}_{xr}\overset{\leftarrow }{x_{t}^{u}}+{\overset{\leftarrow }{W}}_{hr}\overset{\leftarrow }{h_{t-1}}+\overset{\leftarrow }{b_{r}}\right)\\ {\tilde{h}}_{t}=tanh\left(\overset{\leftarrow }{W_{xh}}\overset{\leftarrow }{x_{t}^{u}}\right)+\overset{\leftarrow }{U}\left(\overset{\leftarrow }{r_{t}}⊙\overset{\leftarrow }{h_{t-1}}\right),\overset{\leftarrow }{h_{t}}=\left(1-\overset{\leftarrow }{z_{t}}\right)⊙\overset{\leftarrow }{h_{t-1}}+\overset{\leftarrow }{z_{t}}⊙{\tilde{h}}_{t} \end{array}\right.,backward \end{array}\right.$$

In the above equation, $\vec{h_{t}}$ and $\overset{\leftarrow }{h_{t}}$ represent the forward and backward calculation processes, respectively; ${\overset{\leftarrow }{\Theta }}_{\mathrm{BiGRU}}$ and ${\vec{\Theta }}_{\mathrm{BiGRU}}$ are the parameter sets for the forward and backward processes; $h_{t-1}$ is the hidden status at time t−1; $\tilde{h_{t}}$ is the candidate hidden status at time step t; $\vec{b_{z}}$, $\vec{b_{r}}$ and $\overset{\leftarrow }{b_{z}}$, $\overset{\leftarrow }{b_{r}}$ are the biases during the forward and backward processes; ${\vec{W}}_{xz}$,${\vec{W}}_{hz}$, ${\vec{W}}_{xr}$, ${\vec{W}}_{hr}$, ${\vec{W}}_{xh}$, $\vec{U}$ and ${\overset{\leftarrow }{W}}_{xz}$, ${\overset{\leftarrow }{W}}_{hz}$, ${\overset{\leftarrow }{W}}_{xr}$, ${\overset{\leftarrow }{W}}_{hr}$, ${\overset{\leftarrow }{W}}_{xh}$, $\overset{\leftarrow }{U}$ are the input weights in the forward and backward processes, respectively.

To improve the regression accuracy of the LSTM and BiGRU, we also added mechanisms of attention to the structure of the deep learning network [45]. Suppose the input vectors are the multidimensional feature vectors before the predicted time. The attention coefficient is obtained by calculating the previously hidden layer $h_{i-1}^{\prime }$ and the encoder $h_{j}$ of another LSTM or BIGRU network in the decoder. The attention mechanism is as shown in Equations (13) and (14):

(13) $$e_{ij}=\nu \tanh\left(W\cdot h_{j}+U\cdot h_{i-1}^{\prime }+b\right)$$

(14) $$a_{ij}=\frac{exp\left(e_{ij}\right)}{\sum_{k=t-n}^{t}exp\left(e_{ik}\right)}$$

#### 2.3.3. Health Status Assessment and Classification Modeling

**Figure 3:**
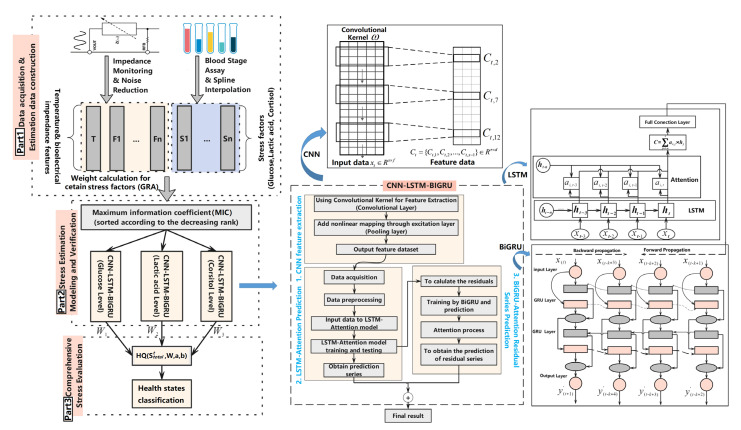
CNN-LSTM-BiGRU-based stress estimation and fuzzy health status classification modeling.

In this section, the CNN-LSTM-BiGRU-based stress evaluation modeling for live fish health evaluation proposes a new direction to comprehensively identify its vitality degree by WBIA measurement. The whole procedure is divided into three parts. The modeling process is shown in Figure 3. 

Part 1 is the acquisition of WBIA-based training data (impedances and phase series) and the biomarkers in fish blood or muscle to construct the stress estimation data set. Moreover, the WBIA signals are harvested with noise removal and smooth processing to establish a relatively ideal training data set. Part 2 is primarily the computation of the MIC between the WBIA signals under the specific frequencies and stress variation series to obtain a well-established training data set. Then, we utilized the CNN-LSTM-BiGRU model to obtain the relevant stress levels, such as glucose, lactate, cortisol, and so forth. In Part 3, we used each stress factor’s estimation to evaluate the final fish health conditions in terms of the weight allocation for each stress indicator and Z-shaped member function.

During the WBIA feature extraction in Figure 3, the convolutional kernel ω acts on the input data $x_{t}\in R^{s\times f}$ of the *t*-th time step, extracting the feature matrix $C_{t}=\left\{C_{t,1},C_{t,2},\cdots ,C_{t,s-1}\right\}\in R^{\tau \times d}$, where f represents the length of the time step; τ is the feature dimensions; X indicates the length of the output feature; S represents the dimension of the output feature, and its size is determined by the filter settings. In this study, the concrete implementation of the fish health classification was constructed using the following steps.

The WBIA signals X were input based on different frequencies, temperature series T, the series of stress factors S, and nutrient variations.

Step 1. Calculate the nutrient-based weight calculation for stress indicators: W=$\left[w_{1},\right.$$\left.w_{2},\cdots ,w_{n}\right]$ using the GRA in Equation (7).
Step 2. Eliminate the gross errors using the Romanovsky criterion in Equation (4).
Step 3. Smooth the WBIA signals using the Savitzky–Golay filter in Equation (5).
Step 4. Calculate the maximum mutual information $MIC\left(X,S\right)$ between X and S according to Equation (8).
Step 5. Select the maximum stress-related WBIA features $X^{*}\left(X^{*}\subset X\right)$ if $MIC\left(X,S\right)\geq 0.9$ to establish a training data set.
Step 6. Extract features of the WBIA series using a CNN: $C_{t}=\left\{C_{t,1},C_{t,2},\cdots ,C_{t,s-1}\right\}$.
Step 7. Utilize LSTM for the estimation of one stress S each time and calculate the residual series $res_{i}$ with an attention mechanism.
Step 8. Regress the residual series $res_{i}$ using the BiGRU network (hidden status: $H_{k}=\left(S^{k};{\vec{\Theta }}_{BiGRU},{\overset{\leftarrow }{\Theta }}_{BiGRU}\right)$) with an attention mechanism using Equations (12)–(14).
Step 9. Input the learning features of the fully connected layer to generate an estimated stress result $\hat{S_{i}\left(t\right)}$ at time point t, and then normalize the i-th stress to obtain $\hat{norm\left(S_{i}\left(t\right)\right)}$.
Step 10. Finally, evaluate the health status $HQ\left(\hat{S_{total}^{t}},W,a,b\right)$ by the total normalized stress at time t with the temperature zone ($T_{low},T_{high}$) and the ZMF adjustment coefficients (a and b).

## 3. Results and Discussion

This section provides a detailed description and discussion of the experimental results of the stress status estimation and health level classification. All these calculations and data visualization were carried out using MATLAB R2022b and Python 3.11.4 with the Scikit-learn 1.3 library as the data analyzing and processing tools. In this study, the evaluation indices are first listed for the forthcoming model’s calculation and its verifications. The training and testing data sets for validation comprised 80% and 20%, respectively.

### 3.1. Evaluation Criterion

In this work, the mean absolute error (MAE), the mean absolute percentage error (MAPE), and the root mean squared error (RMSE) were used to assess the performance of the stress evaluation models. To judge the conditions of the fish health status, indexes such as the accuracy, F1 score, precision, and recall were utilized to measure the final classification performance of the fish health status [46]. The relevant calculations are shown in Equations (15)–(18).

(15) $$Precision=\frac{TP}{\left(TP+FP\right)}\times 100\%$$

(16) $$\;Recall=\frac{TP}{\left(TP+FN\right)}\times 100\%$$

(17) $$Accuracy=\frac{TP+TN}{TP+TN+FP+FN}\times 100\%$$

(18) $$F1-Score=2\frac{\;Precision\times Recall\;}{Precision+Recall\;}$$

### 3.2. WBIA Feature Selection

Physiological biomarkers were discretely and accurately detected to obtain the main trends of fish stress variations. In this work, cubic spline interpolation technology was utilized to complete the missing data and construct a time series-based stress data set that was similar to real stress changes. The stress prediction system established a hybrid deep neural network to complete short-term stress trend estimation and health quality evaluation by utilizing a WBIA feature data series and an interpolated key stress series as the training data set.

**Figure 4:**
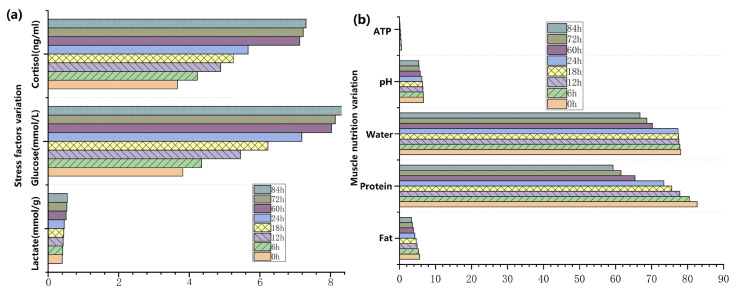
The periodic substance testing of the physiological stresses and muscle nutrients of dormant turbot at different checkpoints under waterless and low-temperature (1–3 °C) conditions, (**a**) Stress factors variation; (**b**) Muscle nutrition variation.

**Figure 5:**
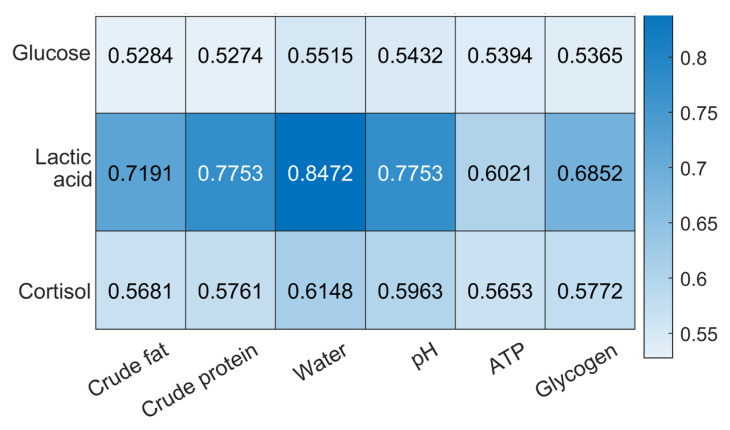
Heatmap of key stress indicators and critical nutrients using GRA.

Figure 4 shows the periodic substance testing of physiological stresses and muscle nutrients of dormant turbot at different checkpoints under waterless and low-temperature (1–3 °C) conditions. Through the above biomarker and nutrient test, the physiological stress levels and muscle nutritional components of the turbot under such adverse conditions were calculated and are shown in Figure 5. Based on the above analysis, we assigned the proper weights for each stress index according to the relationship between the muscle nutrients and physiological stress levels using GRA. Lactate dominated the most important position among the three key stress factors. The weight allocations were $W_{Lactate}$ (0.396), $W_{Glucose}$ (0.290), and $W_{Cortisol}$ (0.314), which were calculated using Equation (19).

The normalization of all stresses is denoted by Equation (19).

(19) $$S_{total}=\sum_{i=1}^{3}\left[W_{i}\times norm\left(S_{i}\right)\right]=W_{Glucose}\times norm\left(G\right)+W_{Lactate}\times norm\left(L\right)+W_{Cortisol}\times norm\left(C\right)$$

where $norm\left(.\right)$ is the normalized function to map the data to a range of [0, 1].

Among the WBIA features, some stress-related features were chosen as the input data set to improve the efficiency of the ML algorithms. Through the MIC calculation between the physiological stress indicators and the WBIA-based features (phase θ, bioimpedance Z), the critical components of the WBIA were calculated and are recorded in Table 2 and Table 3. After the calculation, the values of bioimpedance at frequencies of 80, 90, and 100 and the values in the phase of BIA at frequencies of 70, 80, and 90 were screened out for the forthcoming training and testing using the single or compound ML approaches.

### 3.3. Stress Evaluation Verification

**Figure 6:**
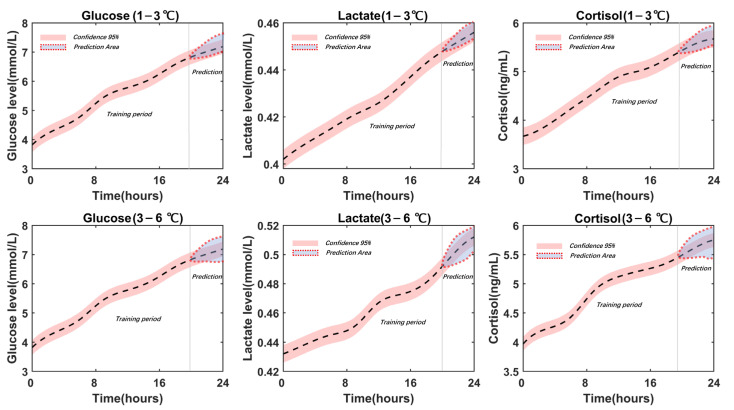
Stress trend evaluation using CNN-LSTM-BiGRU in 24 h under adverse conditions (parameters of BiGRU: layers: 2, neurons: 32, dense layer neurons: 32, fully connected layers: 3, batch size: 32, optimizer: Adam, sliding window length: 15).

The three key stress factors were continuously estimated using the sliding window strategy. The main stress-changing scope was also visualized with a confidence of 95% in the prediction periods. As can be seen in Figure 6, the deviation of the stress level evaluation was relatively small, at 1–3 °C. 

Meanwhile, based on the enclosed predicted area and the real data fitting curves with a confidence of 95%, the prediction deviations increased with the rise in the temperature zone to 3–6 °C. Therefore, the stress assessment was in the ideal expected range when the temperature zone was confined to 1–3 °C.

**Figure 7:**
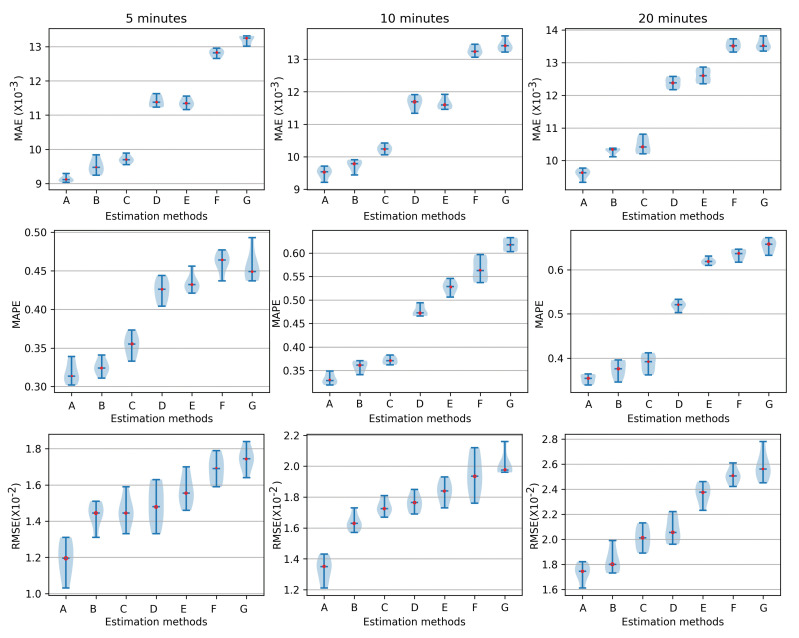
The MAE, MAPE, and RMSE of stress level (glucose, lactate, and cortisol) estimations at different time points (5 min, 10 min, 20 min) in 1–3 °C, A: CNN-LSTM-BiGRU, B: CNN-LSTM, C: CNN-GRU, D: GRU, E: LSTM, F: SVR, G: BP.

**Figure 8:**
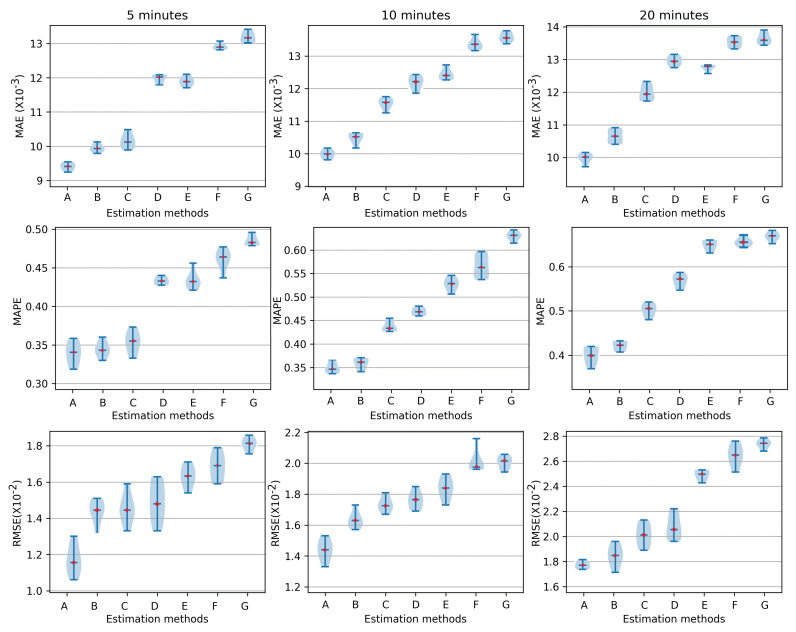
The MAE, MAPE, and RMSE of stress level (glucose, lactate, and cortisol) estimations at different time points (5 min, 10 min, 20 min) in 3–6 °C, A: CNN-LSTM-BiGRU, B: CNN-LSTM, C: CNN-GRU, D: GRU, E: LSTM, F: SVR, G: BP.

After that, we calculated the errors of the stress trend estimation in terms of the WBIA signals using ML approaches under 1–3 °C and 3–6 °C, which are clearly illustrated in Figure 7 and Figure 8, respectively. In the process of the evaluation comparisons, the ML method configuration is clarified as follows: GRU: layers: 2, neurons: 30–40, fully connected layers: 2, dense layers: 10, batch size: 30, optimizer: Adam; LSTM: layers: 2, neurons 30–40, fully connected layers: 2, dense layers: 10, batch size: 30, optimizer: Adam; SVM: kernel function: RBF, loss function: 0.01, C: 10, gamma: 0.3; BP: hidden layers: 9; input layers: 7, learning rate: 0.01. 

With the increase in the estimation steps, the MAE was about 9.06 at 5 min, 9.33 at 10 min, and 9.61 at 20 min. The MAPE was 0.319, 0.339, and 0.359, respectively, and it also clearly showed little deviation in the stress trends at different times compared to the other estimation methods. Regarding the RMSE at 1–3 °C, it individually demonstrated excellent estimation results of 1.03, 1.27, and 1.57. At the same while, the RMSE of CNN-LSTM and CNN-GRU reached about 2.13 and 2.15. Finally, Figure 7 shows that our method exhibited superior performance. Concurrently, in Figure 8, the three deviation indices all increased at different degrees within 3–6 °C. Regarding the proposed approach, the MAE was 9.39, 10.05, and 10.09 at 5 min, 10 min, and 20 min, respectively. The MAPE reached 0.341, 0.349, and 0.402 at the above time points. Finally, the RMSEs of CNN-LSTM-BiGRU were 1.13, 1.43, and 1.76, respectively, which means increased error in the stress evaluation.

### 3.4. Health Fuzzy Evaluation Verification

According to the experimental results and previous studies, the surface and behavioral features of cold dormant live turbot in adverse conditions are listed in Table 4, along with their roughly defined health levels and survival durations.

Based on the health quality mapping function, health levels can also be empirically classified according to the principle of the close or far distance from the most suitable dormant temperature zone. In this research, 1–3 °C was more suitable than 3–6 °C and could better reduce external stimuli and achieve the best cold dormancy status for fish. At a certain health level status, the corresponding total stress was deduced for certain health levels, the mapping relationship of which can be approximately fitted by the ZMF curve with the optimized a, b.The average value $S_{total}^{t}$ accurately reflects the real health status. Additionally, $S_{total}^{t}$ is the invasive accurate biomarker test result and not the estimated results $\hat{S_{total}^{t}}$ of the WBIA-based stress evaluations. Through the test, the survival rates of 25 turbots treated with waterless and dormant preservation were tested, and the survival rates were observed to detect the near-death time points. They had a high survival rate within 60 h, with two dead at 60 h, three dead at 72 h, three dead at 78 h, and three dead at 84 h (survival rate: 56%). We took 84 h as the near-death point to continue this study. In this work, we define the near-death time point as 84 h. Table 5 shows the health level criterion that is expressed by the suggested health score ranges.

**Figure 9:**
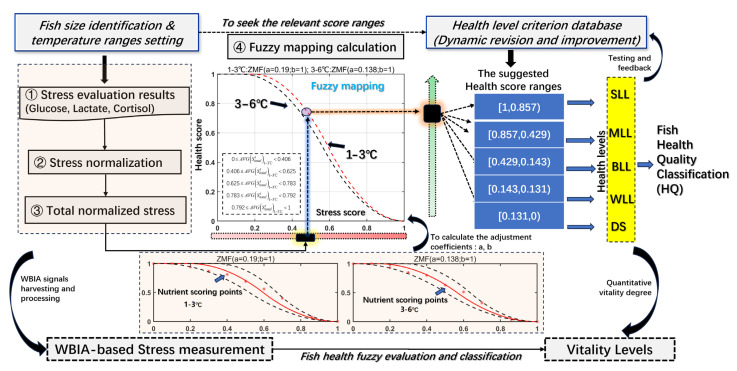
Fish health quality fuzzy classification procedures.

In this section, we assign the health quality (HQ) a number from 0 to 1, which represent the weak live level (WLL) to the strong live level (SLL). The suggested health score range was determined by the surface and behavior features. It is roughly divided into five levels, which express the vitality levels. Regarding parameters a and b, we used the two ZMF curves to envelope the nutrient scoring points in Figure 9 from SLL to DS to deduce the basic curve equations for different temperatures’ HQ classification. Through the above mapping calculation, the turbot health quality was classified by evaluating the stress trends under adverse conditions.

In this study, medium-sized fish were chosen for the health evaluation test. The experiments were carried out at the time points of 24 h and 74 h for the biomarkers test of turbot and compared with the WBIA-based stress evaluation results at 1–3 °C and 3–6 °C to validate the accuracy of the HQ classification using the criteria of the F1 score, precision, accuracy, and recall. Through the final deviation of health classification testing and feedback of the validations of real health indexes (biomarkers in blood and muscle nutrients), the assessment system can make dynamic revisions and improve the HQ criterion database by fundamentally upgrading the HQ classification performance. We calculated the average values as the evaluation and classification results. In the comparative outcomes, the micro-metrics were calculated globally by counting the total true positives, false negatives, and false positives. In addition, the macro-metrics were computed for each label, and we obtained their unweighted mean. This did not take label imbalance into account. The classification verification for the health status evaluation at different temperatures is shown in Table 6.

The final validation results show that the classification using the Z-shaped membership function had the ideal performance at 1–3 °C. The accuracy was about 0.917 after the classified health evaluations. The F1 score was up to about 0.916 on average. The precision (micro) and precision (macro) were 0.917 and 0.938 respectively, which are relatively high scores. The recall (micro, macro) was under suitable temperature conditions and the score reached about 0.917. Meanwhile, the performance slightly decreased when the temperature rose to 3–6 °C. With the increments in the temperature range, uncertainty adversely affected the final health status evaluation. Specifically, the F1 score was reduced to about 0.831 on average. The precision, recall, and accuracy decreased to 0.853, 0.832, and 0.833, respectively. The test outcomes indicate that the increment in the ambient temperature region under adverse conditions may have reduced the performance of the live fish health assessment to varying degrees.

### 3.5. System Evaluation and Suggestions

The evaluation of the health monitoring system for live turbot includes the optimization and improvement of real-time stress data harvesting, preprocessing, and modeling, and health classification before and after deploying WBIA-based sensing and analyzing devices [47,48,49,50]. The evaluation performance and suggestions for the improvement of the proposed monitoring system enabled by deep learning-based stress estimation and fuzzy health mapping calculation are demonstrated in Table 7.

Through analysis of the above experimental results, we first obtained more accurate stress variations by WBIA feature selection and deep learning-based stress evaluation models. Then, the stress-related fish health fuzzy assessments were also sufficiently verified in this evaluation test and achieved relatively excellent accuracy. Regarding the fish health evaluation system and suggestions, we describe the advantages of our designed WBIA-based stress monitoring system in terms of deep learning techniques and give constructive suggestions for its possible improvement.

## 4. Conclusions

In this research, a wearable bioimpedance-based deep learning technique is proposed to conduct fish stress monitoring and stress-related fuzzy health evaluation. For precisely acquiring different stress indexes, closer correlations between the WBIA signals and the stress variations were selected according to their MIC results. Moreover, to minimize the stress assessment errors, some popular ML approaches, such as CNN-LSTM-BiGRU, CNN-LSTM, CNN-GRU, LSTM, GRU, SVR, and BP network, were applied to reduce the deviations. After the experiments, the results were verified by the MAE, MAPE, and RMSE, which show that the CNN-LSTM-BiGRU exhibited superior accuracy compared to the other ML algorithms. Finally, the fuzzy health classification tests were also validated by the F1 score, accuracy, precision, and recall criteria. The classification performance showed a relatively higher accuracy at 1–3 °C than that at 3–6 °C owing to the fewer stress variations under the optimal cold dormancy levels. Therefore, dynamic WBIA-based sensing for fish health assessment using a deep learning algorithm is a significant method to obtain the optimum survival conditions and effectively provides an in-depth theoretical reference for improving the levels of fish vitality detections.

## Figures and Tables

**Table 1:** Stress detection methods and their pros and cons.

Measurement Mode	Detection Means	Pros	Cons	References
Invasive blood stress factors test	Vein blood extraction from fishtail or tail amputation extraction; blood analysis (microplate reader, medical blood analysis equipment)	Rich detection indicators; the comprehensive reflection of fish stress and health status; high accuracy	Injury or fatal to the fish; no in situ-based test; discontinuous; unable to truly reflect fish dynamic health level	[9,10]
Minimally invasive blood factors test	Implantation of biosensors in fish eye interstitial fluid	Dynamically collect changes in blood glucose, sterols, lactate, and cortisol levels of fish	Anesthetize the fish before deploying the sensors; may cause discomfort; may introduce additional stress	[11,12,13]
Non-invasive stress detection	Applying acceleration sensors; multi-gas sensors; fish skin mucus sensors; millimeter-level radar wave-based sensors for health monitoring	Easy sensor deployment and monitoring carrying out; non-invasive and continuous health status detections	Cannot accurately reflect stress and health status; easily influenced by variations in surroundings	[14,15,16,17]

**Table 2:** MIC calculation between stress indicators and bioimpedance of WBIA.

Bioimpedance Z (Frequency: 30–100 KHZ; Interval: 10 KHZ)	Glucose	Lactate	Cortisol
30	0.225	0.378	0.225
40	0.378	0.378	0.378
50	0.558	0.558	0.558
60	0.225	0.558	0.225
70	0.791	0.991	0.791
**80**	**0.991**	**0.991**	**0.991**
**90**	**0.991**	**0.991**	**0.991**
**100**	**0.991**	**0.991**	**0.991**

**Table 3:** MIC calculation between stress indicators and phase of WBIA.

Phase θ (Frequency: 30–100 KHZ; Interval: 10 KHZ)	Glucose	Lactate	Cortisol
30	0.5900	0.5577	0.5900
40	0.5577	0.5900	0.5577
50	0.5900	0.5900	0.5900
60	0.5900	0.5900	0.5900
**70**	**0.9911**	**0.9911**	**0.9911**
**80**	**0.9911**	**0.9911**	**0.9911**
**90**	**0.9911**	**0.9911**	**0.9911**
100	0.4789	0.5789	0.3789

**Table 4:** The surface and behavioral features of turbot health levels under waterless and cold dormant statues in 1–3 °C.

Health Levels	Respiratory Rate (times/min)	Gill Flapping Amplitude	Body Surface Color	Converged Fin Angle	Behavior Changes	Duration (hours)
SLL	16–21	Normal	Normal	Normal	The respiratory rate is lower than normal, with regular intermittent oscillations of the side fins	0–12
MLL	12–15	Slightly reduced	Partially Slightly darkening	Basic	The respiratory rate is even more lower than normal, and the edge fins occasionally oscillate	13–48
BLL	9–11	Reduced	Overall darkening	Normal	Breathing weakly, and the side fins are not swinging	49–72
WLL	6–8	Weak	Overall darkening and Partial graying	Slightly	Intermittent cessation of breathing and with low frequency, body stiffness	73–84
DS	0	Motionless	Overall darkening, large area grayish white	Converged	Repeated stimulation without response, the disappearance of vital signs	Larger than 84

**Table 5:** Health classification criteria at different temperature ranges. (Fish body size: medium; 1–3 °C: a = 0.019, b = 1; 3–6 °C: a = 0.0138, b = 1).

Temperature Ranges	Health Status	Suggested Health Score Ranges	Total Normalized Stress Ranges
1–3 °C	SLL	[1, 0.857)	0 $\leq AVG{\left(S_{total}^{t}\right)}_{1-3{}^{\circ}C}<$ 0.406
	MLL	[0.857, 0.429)	0.406 $\leq AVG{\left(S_{total}^{t}\right)}_{1-3{}^{\circ}C}<$0.625
	BLL	[0.429, 0.143)	0.625 $\leq AVG{\left(S_{total}^{t}\right)}_{1-3{}^{\circ}C}<$ 0.783
	WLL	[0.143, 0.131)	0.783 $\leq AVG{\left(S_{total}^{t}\right)}_{1-3{}^{\circ}C}<$ 0.792
	DS	[0.131, 0)	0.792 $\leq AVG{\left(S_{total}^{t}\right)}_{1-3{}^{\circ}C}<$ 1
3–6 °C	SLL	[1, 0.857)	0 $\leq AVG{\left(S_{total}^{t}\right)}_{3-6{}^{\circ}C}<$0.368
	MLL	[0.857, 0.429)	0.368 $\leq AVG{\left(S_{total}^{t}\right)}_{3-6{}^{\circ}C}<$0.597
	BLL	[0.429, 0.143)	0.597 $\leq AVG{\left(S_{total}^{t}\right)}_{3-6{}^{\circ}C}<$0.767
	WLL	[0.143, 0.131)	0.767 $\leq AVG{\left(S_{total}^{t}\right)}_{3-6{}^{\circ}C}<$0.779
	DS	[0.131, 0)	0.779 $\leq AVG{\left(S_{total}^{t}\right)}_{3-6{}^{\circ}C}<$1

**Table 6:** Verification of health status classification under different temperature ranges.

Temperature Ranges	Criterion	Scores
1–3 °C	Precision (micro)	0.917
	Recall (micro)	0.917
	Precision (macro)	0.938
	Recall (macro)	0.917
	Accuracy	0.917
	F1 score (micro)	0.917
	F1 score (macro)	0.914
3–6 °C	Precision (micro)	0.832
	Recall (micro)	0.833
	Precision (macro)	0.875
	Recall (macro)	0.831
	Accuracy	0.833
	F1 score (micro)	0.833
	F1 score (macro)	0.829

**Table 7:** The improvement and suggestion for WBIA-based fish health monitoring system.

Content	Previous Health Monitoring System	WBIA-Based Health Monitoring System	Suggestion
Acquisition mode of stress factors	Invasive; minimal invasive; destructive	Non-invasive; wearable	Improvement of WBIA sensors’ flexibility and conformability; reducing the deformation interferences
Multiple stress detection	Relatively simple features for stress signal extractions; cannot track various stress indexes at one time	Multiple frequencies and multiple features in WBIA for selections; can track multiple stress indexes at one time	To select more suitable referenced WBIA features
Weights of stress factors	No definition or calculations	To scientifically allocate the weight of the specified stresses	To dynamically assign the weight for each stress factor during the health status monitoring
Stress evaluation and health classification	Less accuracy; relatively simple modeling; less reasonable mapping calculation; only based on blood biomarkers	More accuracy; deep learning-based modeling; continuous fuzzy mapping based on the blood biomarkers and muscle nutrients	To extend the stress factors (not confined to glucose, lactate, and cortisol) for health evaluations

## Data Availability

The data presented in this study are available upon request from the corresponding author.
